# Maternal and neonatal outcomes following SARS-CoV-2 infection in an unvaccinated pregnant cohort: A trimester-specific analysis

**DOI:** 10.1371/journal.pone.0341647

**Published:** 2026-02-23

**Authors:** Rozhan Khezri, Kamran Ebrahimi, Saeedeh Askari, Shayesteh Jahanfar, Fateme Darvish Motevalli, Kourosh Javdani Esfehani

**Affiliations:** 1 Student Research Committee, Iran University of Medical Sciences, Tehran, Iran; 2 Department of Epidemiology, School of Public Health and Safety, Shahid Beheshti University of Medical Sciences, Tehran, Iran; 3 Department of Midwifery and Reproductive Health, School of Nursing and Midwifery, Shahid Beheshti University of Medical Sciences, Tehran, Iran; 4 School of Public Health and Community Medicine, Tufts School of Medicine, Boston, United States of America; 5 Student Research Committee, Department of Medical Laboratory Science, School of Allied Medical Science, Iran University of Medical Science, Tehran, Iran; 6 Department of Emergency Medicine, Emergency Medicine Management Research Center, Health Management Research Institute, Iran University of Medical Sciences, Tehran, Iran; Xiangya Hospital Central South University, CHINA

## Abstract

Adverse pregnancy outcomes are significant public health issues in developing countries. This study aims to evaluate the trimester-specific impact of COVID-19 infection on maternal and neonatal outcomes in a cohort of unvaccinated Iranian women. A multi-center cross-sectional study was conducted between March 21, 2020, and March 21, 2021, involving 217 unvaccinated pregnant women with RT-PCR-confirmed SARS-CoV-2 infection who delivered in hospitals across three counties in Northwest Iran. Participants were stratified by the trimester of COVID-19 diagnosis: first (n = 20), second (n = 87), and third (n = 110). Data on demographics, as well as maternal, obstetric, and neonatal outcomes, were extracted from the National Health System. Statistical analyses included ANOVA, Chi-square, Fisher’s exact and Kruskal-Wallis tests, with post-hoc Bonferroni corrections. A significant association was found between the trimester of infection and the rate of PTB (p = 0.028). Women infected in the third trimester had a substantially higher PTB rate (26.4%) compared to those infected in the second (11.5%) and first (15.0%) trimesters. Neonatal outcomes also varied significantly; APGAR scores at 1 and 5 minutes were lowest in the third-trimester infection group (8.16 ± 0.78 and 8.39 ± 0.80, respectively) compared to the first (9.08 ± 0.88 and 9.03 ± 0.90) and second (8.45 ± 0.71 and 8.79 ± 0.83) trimesters (p < 0.001 for both). Fever was significantly more prevalent in the third trimester (33.6%) than in the first (10.0%, p = 0.013). No vertical transmission or neonatal deaths were recorded. This study demonstrates a clear trimester-specific risk gradient for SARS-CoV-2 infection in unvaccinated pregnant women. Infection during the third trimester was associated with a significantly increased odds of PTB and lower neonatal APGAR scores(1,5). These findings underscore the critical vulnerability of late pregnancy to COVID-19 complications and highlight the importance of trimester-specific vigilance, enhanced antenatal surveillance, and robust vaccination advocacy for pregnant women.

## 1. Introduction

Pandemics of infectious diseases always have diverse indirect and direct effects on public health, particularly for pregnant women. Adverse pregnancy outcomes, including maternal and neonatal mortality and morbidity, constitute a significant and persistent public health challenge, with a disproportionately high burden in developing countries [1]. Despite significant progress in addressing diseases, infectious diseases remain a primary concern in many aspects of public health [2]. In late 2019, COVID-19 rapidly escalated into a global pandemic, profoundly impacting public health worldwide [3]. COVID-19 mortality and morbidity have markedly differed by region, demographic factors, and underlying health conditions [4]. Spatial analysis of COVID-19 in Iran reveals varied incidence and mortality rates across provinces [5]. As of February 2024, there were 7,627,186 confirmed cases and 146,811 deaths, highlighting significant regional disparities in the pandemic’s impact [5].

The pandemic highlighted the necessity of protecting vulnerable populations and ensuring healthcare equity. Despite advancements in vaccines and treatments, new variants such as Omicron underscore the importance of continuous vigilance in public health [6].

Pregnant women and newborns are particularly vulnerable to COVID-19 [7,8]. This vulnerability stems from a constellation of profound physiological adaptations during pregnancy. Immunologically, pregnancy induces a state of modulated immunity, often described as a shift from T-helper 1 (Th1) to T-helper 2 (Th2) dominance, to tolerate the fetus. While beneficial for preventing rejection, this adaptation can simultaneously dampen the immune response to viral pathogens, potentially increasing susceptibility to severe infection [9]. Physically, the growing uterus elevates the diaphragm, leading to reduced functional residual capacity and increased oxygen consumption, which diminishes respiratory reserve. This makes pregnant women less tolerant of pulmonary complications like pneumonia. Furthermore, pregnancy is associated with a state of endothelial dysfunction and a higher risk of hypercoagulability, which may exacerbate the thrombotic complications observed in severe COVID-19 [10]. COVID-19 significantly affects women’s health during pregnancy [8].

They also encounter higher risks of death and complications such as respiratory distress syndrome and sepsis, along with increased rates of pregnancy-induced hypertension and preeclampsia [10]. In addition, Neonatal SARS-Cov-2 infections are uncommon [11], but can occur via vertical transmission [12]. Transplacental transmission was seen in a newborn whose mother was infected in the third trimester [12]. Most infected infants are asymptomatic or have mild symptoms [13,14].

Improving the prevention and management of adverse pregnancy outcomes is crucial for reducing maternal and newborn mortality and morbidity. Although several studies have been conducted in Iran on maternal and neonatal complications and outcomes in pregnant women with COVID-19 [15–17], None have investigated the impact of the timing of COVID-19 infection on these maternal and neonatal outcomes. While previous studies have established an association between COVID-19 in pregnancy and adverse outcomes such as preterm birth (PTB) [18], the impact of the timing of infection remains a critical area for investigation, particularly across diverse populations. Furthermore, many existing studies were conducted in populations with increasing vaccine coverage. This multi-center cross-sectional study aims to assess the trimester-specific impact of COVID-19 infection on maternal and neonatal outcomes among a cohort of unvaccinated Iranian women. By focusing on this unique demographic during a period of no vaccine availability, our research provides a distinct perspective on the natural history of COVID-19 in pregnancy, free from the confounding effects of vaccine-derived immunity, and contributes essential data from a region underrepresented in the global literature.

## 2. Methods

### 2.1. Study design, period, and setting

The present multi-center cross-sectional study was conducted between March 21, 2020, and March 21, 2021, involving all pregnant women with COVID-19 who gave birth in public and private hospitals across three counties (Mahabad, Miandoab, and Bukan) located in the southern region of West Azerbaijan Province in northwest Iran. In this study, COVID-19 infection was confirmed in all pregnant women through positive RT-PCR test results at any stage of pregnancy, regardless of the presence or absence of clinical symptoms. The data were received and accessed for research purposes on March 22, 2021. All data were provided without any information that could identify individual participants, both during the data collection process and after its completion.

### 2.2. SARS-CoV-2 variant context

The study period (March 21, 2020, to March 21, 2021) corresponds to the initial waves of the COVID-19 pandemic in Iran, characterized by the prevalence of the original wild-type virus and the early Alpha (B.1.1.7) variant. Genomic surveillance studies have confirmed that the Delta (B.1.617.2) variant, which is linked to more severe outcomes, was first identified in the country in late 2021 [19,20].

### 2.3. The inclusion and exclusion criteria

The inclusion criteria for participants were:

1) A confirmed positive RT-PCR test for COVID-19 during any stage of pregnancy.
2) Having delivered by the time of the study.
3) No prior receipt of the COVID-19 vaccine.

Participants were excluded if they met either of the following criteria:

1. Had not given birth by the time of the study (n = 44).
2. Experienced a miscarriage (n = 19).
3. Had incomplete medical or health records (n = 0).
4. Had baseline diseases (n = 108).
5. Were on regular medication (n = 50).
6. Had an induced PTB (n = 10)

**Fig 1:**
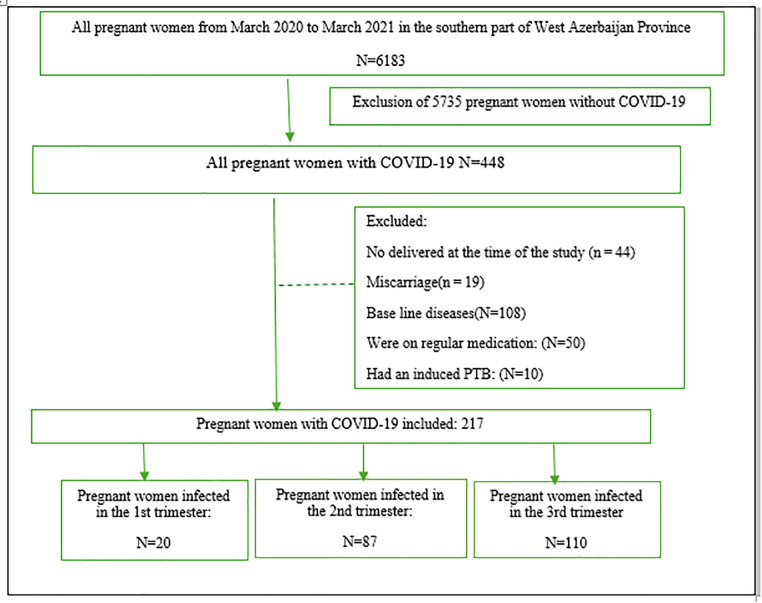
Flowchart illustrating participant inclusion.

The flowchart illustrating the sample selection process is shown in Fig 1.

### 2.4. Data collection

From the initial prenatal visit to the time of delivery, comprehensive data on baseline characteristics, clinical findings, as well as maternal and fetal outcomes, were systematically recorded in the National Health System. In this study, ‘hospitalization’ was defined specifically as admission required for the clinical management of COVID-19 symptoms (e.g., supplemental oxygen, management of severe respiratory symptoms, etc.). Hospital stays that were solely for obstetric indications (e.g., routine delivery, induction of labor) in women with COVID-19 were not counted as COVID-19-related hospitalizations in this study. This specific definition was applied to isolate the hospitalization burden attributable directly to SARS-CoV-2 infection and to avoid the potential confounding of hospitalization duration by delivery-related stays.

The following variables were obtained from electronic medical records in the National Health System: age (years), residency (rural, urban), education (illiterate, < diploma, diploma, > diploma), occupation (housekeeper, employed), symptoms (fever, cough, myalgia, chills, dysgeusia, fatigue, sore throat, headache, dyspnea, anosmia, chest pain, nausea, vomiting), parity (nullipara, multipara), and pre-pregnancy BMI (kg/m²).

### 2.5. Outcome variable

Maternal outcomes included hospitalization, PTB, mode of delivery, and stillbirth. Neonatal outcomes encompassed low birth weight (LBW), APGAR scores at 1 and 5 minutes, COVID-19 positivity, and neonatal death. PTB was defined as babies born alive before completing 37 weeks of pregnancy [21,22]. LBW refers to a newborn with a birth weight under 2500 g [23]. A stillbirth is characterized as the death of a fetus after 20 weeks of pregnancy but before or during delivery [24]. Also, neonatal death was defined as the death of a newborn within the first 28 days of life [25].

### 2.6. Statistical analyses

Categorical variables were presented as frequencies and percentages (N%). Continuous variables were summarized as mean ± standard deviation (SD) or median and interquartile range (IQR), based on their distribution. The normality of continuous data was verified using the Kolmogorov-Smirnov test. The association between categorical variables was assessed using the Chi-square test or Fisher’s exact test. Differences in continuous variables across the three groups were analyzed using the One-way analysis of variance (ANOVA) test for normally distributed data, with post-hoc comparisons (e.g., Bonferroni) conducted when significant differences were found; the Kruskal-Wallis test was used for non-normally distributed data. A p-value less than 0.05 was considered indicative of statistical significance. All statistical analyses were conducted using IBM SPSS Statistics for Windows, version 23.0 (Armonk, NY: IBM Corp).

### 2.7. Ethical consideration

This study was approved by the Research Ethical Review of Urmia University of Medical Sciences (approval number: IR.UMSU.REC.1399.352). The ethics committee of Urmia University of Medical Sciences waived the need for informed consent due to the retrospective nature of the study. All methods have been carried out in accordance with the relevant guidelines and regulations.

## 3. Results

### 3.1. Demographic characteristics of pregnant women by trimester of COVID-19 infection

During the study, 217 pregnant patients with COVID-19 were included in the analysis: 20 (9.2%) were infected in the first trimester, 87 (40.1%) in the second trimester, and 110 (50.7%) in the third trimester. The overall mean age of participants was 29.54 ± 6.32 years. When analyzed by trimester, the mean age was 32.10 ± 6.06 years in the first trimester, 29.29 ± 6.32 years in the second trimester, and 29.28 ± 6.33 years in the third trimester. The majority of participants were housekeepers (93.1%) and resided in urban areas (84.3%). Regarding educational attainment, approximately half of the participants (51.2%) had an education level below diploma, while 23.0% had a diploma and 23.0% had university education. No statistically significant differences were found in demographic characteristics across trimesters (Table 1).

**Table 1:** Demographic Characteristics of Participants by Pregnancy Trimester.

Characteristics	First trimester (N = 20)	Second trimester (N = 87)	Third trimester (N = 110)	Total (N = 217)	P-value
	N(%)/ Mean ± SD	N(%)/ Mean ± SD	N(%)/ Mean ± SD	N(%)/ Mean ± SD	
Age (years)	32.10 ± 6.06	29.29 ± 6.32	29.28 ± 6.33	29.54 ± 6.32	0.156^a^
Residency					
Rural	6 (30.0)	11 (12.6)	17 (15.5)	34 (15.7)	0.156^b^
Urban	14 (70.0)	76 (87.4)	93 (84.5)	183 (84.3)	
Education level					
Illiterate	1 (5.0)	2 (2.3)	3 (2.7)	6 (2.8)	0.366^b^
< diploma	10 (50.0)	45 (51.7)	56 (50.9)	111 (51.2)	
Diploma	4 (20.0)	26 (29.9)	20 (18.2)	50 (23.0)	
> diploma	5 (25.0)	14 (16.1)	31 (28.2)	50 (23.0)	
Occupation					
Housekeeper	17 (85.0)	82 (94.3)	103 (93.6)	202 (93.1)	0.322^b^
Employee	3 (15.0)	5 (5.7)	7 (6.4)	15 (6.9)	

*Note: Continuous data are presented as Mean ± Standard Deviation; Categorical data are presented as Number (%).*

*^a^: P-value for age based on ANOVA test*

*^b^: P-values for categorical variables based on Chi-square test or Fisher’s exact test*

### 3.2. Clinical characteristics of pregnant women with COVID-19

The clinical presentation of COVID-19 symptoms among pregnant women by infection trimester is detailed in Table 2. Analysis of 217 cases revealed that cough (52.1%) and myalgia (40.6%) were the most prevalent symptoms overall. A statistically significant difference was observed in the prevalence of fever across trimesters (p = 0.013), with increasing frequency from first trimester (10.0%) to third trimester (33.6%). No other symptoms showed significant variations across trimesters. The mean BMI across all participants was 27.04 ± 4.68 kg/m², falling within the overweight category according to WHO classification, with no statistically significant differences observed among trimesters (p = 0.586). The majority of symptoms including sore throat, dyspnea, chest pain, fatigue, chill, headache, nausea/vomiting, anosmia, and dysgeusia demonstrated comparable distribution patterns regardless of the trimester of infection.

**Table 2:** Clinical characteristics and BMI of pregnant women by trimester of COVID-19 infection (N = 217).

Characteristics	First trimester (N = 20)	Second trimester (N = 87)	Third trimester (N = 110)	Total (N = 217)	P-value
	N(%)/ Mean ± SD	N(%)/ Mean ± SD	N(%)/ Mean ± SD	N(%)/ Mean ± SD	
BMI (kg/m²)	28.08 ± 5.30	26.94 ± 4.42	26.93 ± 4.79	27.04 ± 4.68	0.586^a^
Fever					
No	18 (90.0)	71 (81.6)	73 (66.4)	162 (74.7)	0.013^b^
Yes	2 (10.0)	16 (18.4)	37 (33.6)	55 (25.3)	
Sore throat					
No	15 (75.0)	47 (54.0)	59 (53.6)	121 (55.8)	0.191^b^
Yes	5 (25.0)	40 (46.0)	51 (46.4)	96 (44.2)	
Cough					
No	10 (50.0)	40 (46.0)	54 (49.1)	104 (47.9)	0.893^b^
Yes	10 (50.0)	47 (54.0)	56 (50.9)	113 (52.1)	
Dyspnea					
No	17 (85.0)	82 (94.3)	98 (89.1)	197 (90.8)	0.297^b^
Yes	3 (15.0)	5 (5.7)	12 (10.9)	20 (9.2)	
Chest pain					
No	20 (100.0)	84 (96.6)	103 (93.6)	207 (95.4)	0.367^b^
Yes	0 (0.0)	3 (3.4)	7 (6.4)	10 (4.6)	
Fatigue					
No	16 (80.0)	75 (86.2)	97 (88.2)	188 (86.6)	0.606^b^
Yes	4 (20.0)	12 (13.8)	13 (11.8)	29 (13.4)	
Myalgia					
No	13 (65.0)	48 (55.2)	68 (61.8)	129 (59.4)	0.557^b^
Yes	7 (35.0)	39 (44.8)	42 (38.2)	88 (40.6)	
Chill					
No	17 (85.0)	67 (77.0)	82 (74.5)	166 (76.5)	0.592^b^
Yes	3 (15.0)	20 (23.0)	28 (25.5)	51 (23.5)	
Headache					
No	17 (85.0)	58 (66.7)	74 (67.3)	149 (68.7)	0.254^b^
Yes	3 (15.0)	29 (33.3)	36 (32.7)	68 (31.3)	
Nausea or vomiting					
No	17 (85.0)	77 (88.5)	99 (90.0)	193 (88.9)	0.795^b^
Yes	3 (15.0)	10 (11.5)	11 (10.0)	24 (11.1)	
Anosmia					
No	12 (60.0)	53 (60.9)	73 (66.4)	138 (63.6)	0.689^b^
Yes	8 (40.0)	34 (39.1)	37 (33.6)	79 (36.4)	
Dysgeusia					
No	13 (65.0)	60 (69.0)	78 (70.9)	151 (69.6)	0.858^b^
Yes	7 (35.0)	27 (31.0)	32 (29.1)	66 (30.4)	

*Note: Continuous data are presented as Mean ± Standard Deviation; Categorical data are presented as Number (%).*

*^a^: P-value based on ANOVA test.*

*^b^: Chi-square test or Fisher’s exact test*

*BMI = Body Mass Index.*

### 3.3. Maternal outcomes according to the trimester of COVID-19 infection

Maternal characteristics and obstetric outcomes were analyzed according to the trimester of COVID-19 infection among the 217 pregnant women included in the study. The overall mean gestational age at delivery was 38.13 ± 2.43 weeks, with no statistically significant difference observed across trimesters (p = 0.648). A significant association was found between the trimester of infection and PTB rates (p = 0.028), with the highest incidence observed following third-trimester infection (26.4%) compared to first (15.0%) or second (11.5%) trimester infections. The median duration of hospitalization was 0 days for all trimesters, with no significant differences found using either the Kruskal-Wallis test (p = 0.387) or the Median test (p = 0.712). No significant associations were detected between the trimester of infection and mode of delivery, or stillbirth rates. There were no maternal deaths recorded in any of the groups. These outcomes are summarized in Table 3.

**Table 3:** Maternal characteristics and obstetric outcomes according to the trimester of COVID-19 infection (N = 217).

Characteristics	First trimester (N = 20)	Second trimester (N = 87)	Third trimester (N = 110)	Total (N = 217)	P-value
Hospitalization (days)					
Mean ± SD	1.23 ± 2.83	1.23 ± 2.83	1.23 ± 2.83	1.23 ± 2.83	–
Median (IQR)	0.00 (0.00)	0.00 (0.00)	0.00 (0.00)	0.00 (0.00)	0.387^a^
Parity, N(%)					
Nullipara	4 (20.0)	18 (20.7)	14 (12.7)	36 (16.6)	0.300^b^
Multipara	16 (80.0)	69 (79.3)	96 (87.3)	181 (83.4)	
Gestational age (weeks)					
Mean ± SD	38.30 ± 2.16	38.29 ± 2.40	37.98 ± 2.51	38.13 ± 2.43	0.648^c^
PTB, N(%)					
No	17 (85.0)	77 (88.5)	81 (73.6)	175 (80.6)	0.028^b^
Yes	3 (15.0)	10 (11.5)	29 (26.4)	42 (19.4)	
Mode of delivery, N(%)					
Vaginal	8 (40.0)	41 (47.1)	59 (53.6)	108 (49.8)	0.435^b^
Cesarean section	12 (60.0)	46 (52.9)	51 (46.4)	109 (50.2)	
Stillbirth, N(%)					
No	19 (95.0)	86 (98.9)	108 (98.2)	213 (98.2)	0.513^b^
Yes	1 (5.0)	1 (1.1)	2 (1.8)	4 (1.8)	
Maternal death	0 (0.0)	0 (0.0)	0 (0.0)	0 (0.0)	–

*Note: Continuous data are presented as Mean ± Standard Deviation or Median (IQR); Categorical data are presented as Number (%).*

*^a^: P-value from Kruskal-Wallis test*

*^b^: P-value from Chi-square test or Fisher’s exact test*

*^c^: P-value from ANOVA test.*

*IQR = Interquartile Range.*

### 3.4. Neonatal outcomes by infection trimester

Neonatal outcomes by infection trimester are summarized in Table 4. The mean birth weight of neonates born to women infected during the first, second, and third trimesters was 3185.00 ± 650.73, 3045.98 ± 561.31, and 2963.64 ± 529.80 grams, respectively (p = 0.215). The mean APGAR score at minute one for neonates showed statistically significant differences across trimesters, with scores of 9.08 ± 0.88, 8.45 ± 0.71, and 8.16 ± 0.78 for first, second, and third trimesters, respectively (p < 0.001). Similarly, APGAR scores at minute five also demonstrated significant differences, with means of 9.03 ± 0.90, 8.79 ± 0.83, and 8.39 ± 0.80 across the three trimesters (p < 0.001).

**Table 4:** Neonatal outcomes by infection trimester (N = 217).

Characteristics	First trimester (N = 20)	Second trimester (N = 87)	Third trimester (N = 110)	Total (N = 217)	P-value
Birthweight (gram), Mean ± SD	3185.00 ± 650.73	3045.98 ± 561.31	2963.64 ± 529.80	3017.05 ± 555.64	0.215
APGAR (1 min), Mean ± SD	9.08 ± 0.88	8.45 ± 0.71	8.16 ± 0.78	8.36 ± 0.80	<0.001^a^
APGAR (5 min), Mean ± SD	9.03 ± 0.90	8.79 ± 0.83	8.39 ± 0.80	8.61 ± 0.85	<0.001^a^
LBW, n (%)	3 (15.0)	13 (14.9)	18 (16.4)	34 (15.7)	0.960
Stillbirth, n (%)	1 (5.0)	1 (1.1)	2 (1.8)	4 (1.8)	0.513
Infant death, n (%)	0 (0)	0 (0)	0 (0)	0 (0)	–

* Note: Continuous data are presented as Mean ± Standard Deviation; Categorical data are presented as Number (%).

*^a^: Statistically significant at p < 0.05*

*Birthweight, Apgar1, and Apgar5 were analyzed using one-way ANOVA with Bonferroni post-hoc test.*

Table 5 showed that post-hoc analysis with Bonferroni correction revealed a clear temporal gradient in Apgar scores. For Apgar1, pairwise comparisons showed significantly higher scores in first versus second trimester (mean difference = 0.621, 95% CI [0.167, 1.075], p = 0.003), first versus third trimester (mean difference = 0.921, 95% CI [0.475, 1.366], p < 0.001), and second versus third trimester (mean difference = 0.300, 95% CI [0.037, 0.562], p = 0.019). Similarly, for Apgar5, significant differences emerged between first and third trimesters (mean difference = 0.634, 95% CI [0.153, 1.115], p = 0.005) and between second and third trimesters (mean difference = 0.396, 95% CI [0.113, 0.680], p = 0.003), though no significant difference was observed between first and second trimesters (p = 0.731).

**Table 5:** Bonferroni post-hoc analysis of significant pairwise comparisons.

Dependent Variable	Comparison	Mean Difference (95% CI)	Standard Error	P-value
Apgar1	1st vs 2nd Trimester	0.621 (0.167 to 1.075)	0.188	0.003^a^
	1st vs 3rd Trimester	0.921 (0.475 to 1.366)	0.185	<0.001^a^
	2nd vs 3rd Trimester	0.300 (0.037 to 0.562)	0.109	0.019^a^
Apgar5	1st vs 3rd Trimester	0.634 (0.153 to 1.115)	0.199	0.005^a^
	2nd vs 3rd Trimester	0.396 (0.113 to 0.680)	0.118	0.003^a^

*a: Statistically significant at p < 0.05*

## 4. Discussion

### 4.1. Main findings

This multi-center cross-sectional study, conducted among a unique cohort of unvaccinated Iranian women during the initial wave of the pandemic, provides critical evidence on the trimester-specific impact of SARS-CoV-2 infection. This study reveals three principal findings. First, and most significantly, the timing of infection is a crucial determinant of PTB, with a pronounced risk gradient: women infected in the third trimester experienced a PTB rate of 26.4%, which is substantially higher than the 11.5% and 15.0% observed in the second and first trimesters, respectively. Second, while no vertical transmission was detected, neonatal condition at birth, as measured by Apgar scores at 1 and 5 minutes, was significantly lower in infants born to mothers with third-trimester infection compared to those infected earlier in pregnancy. Third, the clinical presentation and severity of COVID-19 varied with gestational age, evidenced by a higher prevalence of fever and a trend towards longer hospitalization durations in women infected during the third trimester. These findings underscore that late-pregnancy COVID-19 infection poses the greatest threat to both the course of pregnancy and immediate neonatal well-being in unvaccinated populations.

### 4.2. Comparison with the literature

Our core finding that third-trimester COVID-19 infection significantly elevates the risk of PTB resonates strongly with the global literature, thereby reinforcing the external validity of this association. A large population-based cohort study from Israel by Fallach et al. (2022) provided compelling evidence for this temporal relationship; infection during the third trimester, particularly after 34 weeks of gestation, was a strong independent risk factor for spontaneous preterm delivery [26]. Similarly, a comprehensive study from the United States by Stock et al. (2022) reported that pregnancies complicated by SARS-CoV-2 infection, especially during the Delta variant period, were associated with increased rates of stillbirth and PTB, with the latter being more common when infection occurred closer to term [7]. The pathophysiological rationale for this is well-grounded in the physiological adaptations of pregnancy. The third trimester is characterized by a state of relative immunodysregulation, decreased functional residual lung capacity, and a heightened pro-thrombotic state [10] SARS-CoV-2 infection exacerbates these conditions, potentially triggering a systemic inflammatory response that can lead to placental dysfunction and subsequently, uterine irritability or medical indications for early delivery [10].

The strength of our study lies in its demonstration of this risk within a distinct demographic context. The overall PTB incidence of 20.3% in our cohort is markedly higher than the pre-pandemic baseline rates of 9.2% to 12.4% reported for Iran in previous epidemiological studies [17,22]. This stark disparity suggests that SARS-CoV-2 infection conferred a substantial added burden on pregnancy outcomes in our population. Our findings are consistent with the international meta-analyses and cohort studies that established COVID-19 as a significant risk factor for PTB [7]. However, the magnitude of the effect we observed in the third trimester appears high. This may be attributed to the unique characteristics of our study population: all participants were unvaccinated.The absence of vaccine-induced immunity likely resulted in more severe maternal infection, which is a known driver of iatrogenic PTB [27].

Regarding neonatal outcomes, our finding of significantly lower Apgar scores at 1 and 5 minutes following third-trimester infection is a nuanced and important contribution. While many studies, including a meta-analysis by Neef et al (2021), have reported that most neonates born to infected mothers are asymptomatic and have good outcomes [13], few have performed a detailed trimester-based analysis of Apgar scores [14]. Our results suggest that even in the absence of confirmed vertical transmission or severe neonatal disease, the intrauterine environment of a mother with active late-pregnancy COVID-19 may be suboptimal, potentially due to subclinical placental insufficiency or transient maternal hypoxia affecting fetal oxygenation. This is supported by studies that have identified placental damage and inflammation in COVID-19 positive pregnancies, which can compromise fetal reserve and manifest as lower Apgar scores without progressing to stillbirth or neonatal death [11]. It is critical to interpret these findings in context; the mean 5-minute Apgar scores, while statistically lower, remained above 8 across all groups, indicating generally good neonatal condition. Nevertheless, the statistically significant decline warrants attention as a marker of fetal stress. Additionally, none of the newborns tested positive for COVID-19 immediately after birth. Similarly, a meta-analysis revealed that most neonates born to mothers with COVID-19 did not show any signs of positive infection [13].

Our analysis of maternal symptoms and hospitalization aligns with the existing understanding of COVID-19 severity in pregnancy. The trend of increasing hospitalization duration from the first to the third trimester, though not statistically significant in median values, reflects a pattern observed by Schell et al. (2022) and Sief et al (2023), who reported that disease progression and severity were more pronounced with later diagnosis [2,27]. The significant increase in fever prevalence during the third trimester in our cohort is a clinically relevant indicator of a more robust systemic inflammatory response, which could be a key mediator linking late-pregnancy infection to adverse outcomes like PTB [10]. The higher prevalence of nausea and vomiting in the first trimester is most parsimoniously explained by physiological pregnancy changes rather than a specific effect of SARS-CoV-2. Furthermore, the prevalence of gastroesophageal reflux disease (GERD) increases significantly as pregnancy progresses [28], which could confound the attribution of symptoms like nausea and vomiting to COVID-19 in later trimesters. While our data did not specifically differentiate GERD from other causes of nausea/vomiting, this physiological progression reinforces the importance of interpreting symptom data within the context of gestational age.

### 4.3. Translational implications for public health

The findings from this study carry significant translational implications for public health policy, clinical practice, and health systems planning, particularly in low- and middle-income countries (LMICs) with similar unvaccinated or under-vaccinated pregnant populations.

- **Enhanced antenatal surveillance and triage:** Our results argue for a trimester-stratified approach to managing COVID-19 in pregnancy. Public health guidelines should emphasize that pregnant women, especially those in their third trimester, constitute a high-risk group for severe COVID-19 and its complications. Symptomatic women presenting in late pregnancy should be prioritized for clinical assessment, monitoring for signs of preterm labor, and may require a lower threshold for hospitalization to manage potential respiratory compromise or disease progression.
- **Vaccination advocacy and prioritization:** Perhaps the most powerful public health implication is the reinforced critical importance of COVID-19 vaccination for pregnant women. Our data, derived from a pre-vaccination cohort, illustrate the “natural history” of the disease and its significant risks. These findings can be leveraged in public health campaigns to counteract vaccine hesitancy by providing clear, local evidence of the dangers of COVID-19 in pregnancy. Pregnant women should be actively prioritized in vaccination rollouts, with messaging that emphasizes protection against PTB and severe maternal morbidity, particularly during the high-risk third trimester.
- **Resource allocation and health system preparedness:** The increased rates of PTB and longer hospital stays associated with third-trimester infections have direct implications for resource allocation. Hospitals serving pregnant populations must ensure adequate capacity in obstetric wards, neonatal intensive care units (NICUs), and isolation facilities to manage potential surges in preterm deliveries and infected mothers. Prenatal care programs should integrate COVID-19 prevention education, early testing, and clear referral pathways for infected pregnant women.
- **Long-term follow-up and research:** The finding of lower Apgar scores, while not indicating severe immediate morbidity, raises questions about potential long-term neurodevelopmental outcomes. Public health systems should consider establishing registries for long-term follow-up of infants born to mothers with COVID-19. Further research is needed to investigate the mechanisms of placental damage and fetal impact, which could inform future therapeutic interventions.
- **Implications for the inclusion of pregnant women in clinical research:** The quantifiable and trimester-specific risks demonstrated in our findings, namely, the significantly elevated incidence of spontaneous PTB and the graded decline in neonatal Apgar scores associated with third-trimester SARS-CoV-2 infection highlight a critical imperative for the systematic inclusion of pregnant individuals in clinical trials for vaccines and therapeutics. The historical paradigm of excluding this population from research, ostensibly for fetal protection, has paradoxically engendered a critical evidence deficit, rendering them therapeutic orphans when interventions are deployed at scale [29–31]. Our data, which precisely delineate the severe maternal and neonatal morbidity in an immunologically naive cohort, provide empirical weight to the ethical argument against this exclusion. A rigorous risk-benefit analysis must now explicitly factor in the significant baseline risks of the disease itself, which our study helps to define. Consequently, the principle of justice in research necessitates the proactive enrollment of pregnant persons to generate safety, immunogenicity, and efficacy data relevant to their distinct pathophysiology [7,30]. Integrating pregnant women into clinical trial frameworks from the outset is not merely a logistical consideration but a moral and scientific necessity to ensure they are protected by evidence-based interventions in future public health emergencies, rather than remaining a vulnerable and understudied population.

## 5. Strengths and limitations

The primary strength of this study is that its robust sample size allows for a thorough analysis of maternal and neonatal outcomes. Additionally, it is a multi-center design, spanning three counties, which enhances the findings’ generalizability. A further strength of this study lies in its contribution to understanding the impact of SARS-CoV-2 in a resource-limited setting prior to vaccine availability. Our findings, derived from an unvaccinated cohort in Iran, provide critical insights into the natural history of COVID-19 in pregnancy, unobscured by the effects of immunization. This context is particularly relevant for many low- and middle-income countries (LMICs), which often experience delays in accessing novel medical countermeasures during global health emergencies. consequently, the data presented here offer a valuable benchmark of the maternal and neonatal risks associated with the unmitigated spread of a novel pathogen in a pregnant population. These results can inform evidence-based public health planning and clinical guidelines, emphasizing the need for targeted protective strategies for pregnant women in the preparedness plans of LMICs, including Iran, for future pandemics.

This study has several limitations. Firstly, as it employs a cross-sectional design, it does not allow for establishing causal relationships. Secondly, the absence of a matched control group of pregnant women without COVID-19 infection limits our ability to make direct comparisons and fully quantify the attributable risk of the virus on the observed outcomes. Thirdly, the RT-PCR test for COVID-19 used in the study may have produced false-positive results (9). Another limitation of our study is the lack of data on Neonatal Intensive Care Unit (NICU) admissions, which would have provided a further dimension to the neonatal outcomes. Future prospective studies should include this important metric to fully characterize the short-term morbidity associated with in-utero SARS-CoV-2 exposure.

## 6. Conclusion

This multi-center cross-sectional study demonstrates a significant association between the trimester of SARS-CoV-2 infection and specific adverse pregnancy outcomes in a cohort of unvaccinated Iranian women. Our analysis reveals a clear trimester-specific risk gradient, with third-trimester infection emerging as the most critical period for adverse outcomes. The key findings indicate that infection during the third trimester is significantly associated with increased rates of PTB (26.4% vs 11.5% in second trimester, p = 0.028), longer hospitalization durations, and significantly lower Apgar scores at both 1 and 5 minutes compared to infections acquired earlier in gestation.

These results substantially advance our understanding of COVID-19’s impact on pregnancy by precisely quantifying the temporal relationship between infection timing and adverse outcomes. The finding of progressively lower Apgar scores with later infection timing, even in the absence of vertical transmission, suggests potential subclinical effects on fetal wellbeing that warrant further investigation. From a clinical perspective, these findings support implementing trimester-stratified management protocols for COVID-19 in pregnancy, with enhanced surveillance and lower thresholds for intervention for women infected during their third trimester. The significant burden of morbidity observed in this unvaccinated cohort underscores the continued importance of COVID-19 vaccination during pregnancy as a crucial preventive strategy. Future research should prioritize prospective longitudinal studies with integrated placental pathology to elucidate the mechanisms underlying the observed trimester-specific effects and to establish causal relationships between infection timing and adverse neonatal outcomes.

## Supporting information

S1 Data Data. (SAV)
